# Scoliosis in osteogenesis imperfecta: identifying the genetic and non-genetic factors affecting severity and progression from longitudinal data of 290 patients

**DOI:** 10.1186/s13023-023-02906-z

**Published:** 2023-09-20

**Authors:** Peikai Chen, Yapeng Zhou, Zhijia Tan, Yunzhi Lin, Daniel Li-Liang Lin, Jingwei Wu, Zeluan Li, Hiu Tung Shek, Jianbin Wu, Yong Hu, Feng Zhu, Danny Chan, Kenneth Man-Chee Cheung, Michael Kai-Tsun To

**Affiliations:** 1 Department of Orthopedics and Traumatology, The University of Hong Kong – Shenzhen Hospital (HKU-SZH), Shenzhen, 518053 Guangdong China; 2 School of Biomedical Sciences, The University of Hong Kong, Pok Fu Lam, Hong Kong; 3 Department of Orthopedics and Traumatology, The University of Hong Kong, Pok Fu Lam, Hong Kong; 4 The Artificial Intelligence and Big Data (AIBD) Lab, The University of Hong Kong - Shenzhen Hospital, Shenzhen, 518053, Guangdong, China

**Keywords:** Scoliosis, Osteogenesis imperfecta, Genetics, Progression rate, Logistic regression, Multivariate regression

## Abstract

**Background:**

Scoliosis is widely prevalent among osteogenesis imperfecta (OI) patients, and is progressive with age. However, factors affecting scoliosis in OI are not well known.

**Methods:**

We retrospectively retrieved longitudinal radiographic and clinical records of consecutive OI patients seeking treatments at our hospital from 2014 to 2022, graded their pre-operative spinal conditions into four outcome groups, estimated their progression rates, and descriptively and inferentially analyzed the genetic and non-genetic factors that may affect the outcomes and progression rates.

**Results:**

In all, 290 OI patients met the inclusion criteria, where 221 had genetic records. Of these 221, about 2/3 had mutations in *COL1A1* or *COL1A2*, followed by mutations in *WNT1* (9.0%), *IFITM5* (9.0%) and other OI risk genes. With an average age of 12.0 years (interquartile range [IQR] 6.9–16.1), 70.7% of the cohort had scoliosis (Cobb angle > 10°), including 106 (36.5%) mild (10°–25°), 40 (13.8%) moderate (25°–50°), and 59 (20.3%) severe (> 50°) scoliosis patients. Patients with either *COL1A1* and *COL1A2* were strongly biased toward having mild or no scoliosis, whereas patients with mutations in *IFITM5*, *WNT1* and other recessive genes were more evenly distributed among the four outcome grades. Lower-limb discrepancy, bone mineral density (BMD) and age of first drug used were all significantly correlated with severity outcomes. Using multivariate logistic regression, we estimated that each year older adds an odds ratio of 1.13 (95% confidence interval [CI] 1.07–1.2) in progression into advanced stages of scoliosis. We estimated a cohort-wide progression rate of 2.7 degrees per year (95% CI 2.4–3.0). Early-onset patients experienced fast progressions during both infantile and adolescent stages. Twenty-five of the 59 (42.8%) patients with severe scoliosis underwent spinal surgeries, enjoying an average Cobb angle reduction of 33° (IQR 23–40) postoperatively.

**Conclusion:**

The severity and progression of scoliosis in osteogenesis imperfecta were affected by genetic factors including genotypes and mutation types, and non-genetic factors including age and BMD. As compared with *COL1A1*, mutations in *COL1A2* were less damaging while those on *IFITM5* and other recessive genes conferred damaging effects. Progression rates were the fastest in the adolescent adult age-group.

**Supplementary Information:**

The online version contains supplementary material available at 10.1186/s13023-023-02906-z.

## Introduction

Osteogenesis imperfecta (OI) is a rare congenital musculoskeletal disorder caused by mutations in ~ 20 genes related to type I collagen synthesis, with impact on osteoblast differentiation and mineralization in bone [1, 2]. OI patients present with low bone density, high fracture rates, long bone deformity, scoliosis and a wide array of other symptoms [3, 4]. Current treatments, including bone density enhancement and orthopedic corrections [5], do not fundamentally cure the condition, causing considerable burdens on affected individuals and society.

Traditionally, OI was clinically grouped into four subtypes (I, II, III and IV) based on the Sillence classification, with type I being mildest, followed by type IV and then type III. Type II is perinatally lethal and thus most serious [6]. The modified classification includes the original four subtypes and a new type V that has unique clinical phenotypes and is uniquely caused by a single-point mutation in the 5’-UTR of *IFITM5* (c. -14C > T) [7, 8]. With rapid technical advances in the past decade, OI is also classified genetically. Patients with *COL1A1/2* and *IFITM5* mutations remain categorized according to the Sillence scheme, while those with mutations in the other 17 genes are subtyped from VI (OMIM #613982) to XXII (OMIM #619795) [4].

Scoliosis, which affects physical mobilities and cardiopulmonary functions, is a form of lateral deformity of the spine (defined as Cobb angle > 10°), categorized into idiopathic (i.e. unknown causes), congenital and neuromuscular subtypes [9]. Scoliosis is commonly found in OI patients [10], with an estimated prevalence of around 50% [11, 12]. Scoliosis in OI is progressive, with an estimated Cobb angle increase of 2.3°–2.6° per year [11–14]. The causes of OI scoliosis are not clear. However, genetics [15], age, gender, drug treatment [16], bone density [17], Sillence classification, and deformities in the limbs or joints [18] are implicated as potential risk factors. Previous efforts studied the risk factors associated with OI scoliosis and suggested potential treatment strategies. Unfortunately, these studies had either small sample sizes [16] or incomplete genotypes [12].

To delineate the relationship between OI scoliosis and potential risk factors, we retrieved the medical records of all consecutive OI patients from 2014 to 2022 seeking treatments at our hospital. Genetic testing results were included where possible, with affected genes covering *COL1A1/A2*, *IFITM5*, *WNT1*, *SERPINF1*, *FKBP10*, etc. Genetic inheritance (AD/AR) and mutation types (qualitative or quantitative) were also documented. We also retrieved information of skeletal maturity, bone density, drug history, Sillence subtypes, and conditions in the limbs. Based on the Cobb angle of the major curves, we stratified the patients into four severity grades: non-scoliotic, mild, moderate and severe [19]. We then performed univariate and multivariate analyses between the independent variables and the severity outcomes. We estimated the progression rates based on longitudinal radiographs and conducted multiple linear regression to identify associated factors.

## Methods

### Samples and materials

Records of all patients diagnosed with osteogenesis imperfecta (OI) in our hospital from August 2014 to November 2022 were retrieved for the current study (n = 308). Eighteen patients without radiographs of the spine were excluded. For each of the remaining 290 patients with spine radiographs, sitting or standing Cobb angles at the thoracic, thoracic-lumbar (TL), and lumbar regions were measured. Each patient may thus have up to three curves, of which the one with the maximum Cobb angle was designated the major curve. All Cobb angles were measured by three experienced pediatric orthopedic surgeons (YPZ, YZL and DLLL). Each patient had one or multiple spinal radiograph follow-ups. Data from the follow-up with maximum major curve Cobb angle was used to grade scoliosis severity. Patients with Cobb angle below 10° were considered non-scoliotic, while those with preoperative Cobb angles between 10°–25°, 25°–50° and > 50° were considered mild, moderate and severe scoliosis, respectively.

Drug treatment history, including the dates and types of anti-osteoporotic agents, was retrieved. Patients were contacted for confirmation of first date of drug treatment and the menarches for female patients. The BMD data were retrieved from the Discovery DXA system (Hologic Inc., Massachusetts) at our hospital. The total hip and lumber regions (L1–L4) BMDs were used. Weight and height corresponding to each BMD measurement were also collected.

The patients were clinically categorized based on the radiographic features, BMD reports and drug treatment histories according to the criteria of modified Sillence classification [7], where patients were labelled as types I–V. At least two of our pediatric orthopedic surgeons (YPZ, YZL, DLLL, JWW and LF) were involved in independently rating each patient. In case of ambiguity, three or more physicians were invited to rate for a final consensus. MKTT reviewed and approved the final subtyping results.

### Genetic sequencing

Targeted amplicon sequencing was performed on 221 out of the 290 patients. Nineteen OI causative genes (including *COL1A1, COL1A2, IFITM5, SERPINF1, CRTAP, P3H1, PPIB, SERPINH1, FKBP10, BMP1, SP7, TMEM38B, WNT1, CREB3L1, SPARC, TENT5A, MBTPS2, MESD, KDELR2*) and 5 OI related genes (*PLOD2, P4HB, SEC24D, PLS3, LRP5*) were included in the sequencing panel. Sequencing results from 167 out of 221 patients were previously published by our team [2], while the other 54 were newly tested cases (Additional file 2). According to the same criteria [2], the single nucleotide variants in *COL1A1* and *COL1A2* were classified into variants with a qualitative impact (missense) and those with a quantitative (variants leading to stop codons, splicing, or frameshift) impact.

### Statistics

Depending on evidence of onset ages, the scoliosis group was divided into early-onset (EOS), late-onset (LOS), and ‘unknown’, based on radiographic evidence and a recommended consensus of demarcation point at the age of 10 [20]. The EOS group had radiographic evidence of scoliosis before the age of 10. The LOS group had radiographic evidence of no scoliosis up to 10 or above, and that of scoliosis afterwards. The “unknown group” refers to the scoliosis patients, information of whose spinal condition before the age of 10 years old is missing. This group may thus contain both early and late onset patients.

To estimate the progression rates, we first excluded the 40 postoperative data-points in the 25 patients with surgical intervention on the spines, and obtained 606 preoperative Cobb angles of major curves in 290 patients. The progression rate was calculated as (angle difference)/(age difference in years) between successive data-points of the same patient. For the first data-point in each patient, this was calculated as (first angle)/(first age), which effectively assumed a constant progression rate since birth. The mid-point of the two ages associated with the two adjacent radiographs was used as the age corresponding with the progression rate estimate.

The statistical analyses were conducted on the R platform (version 4.0.0). For dichotomous quantitative variables, Student's *t*-test was used for univariate analyses. For those with multiple groups, one-way ANOVA was used. For categorical variables, Pearson’s Chi-squared test was used. For multivariate analyses with dichotomous dependent variables, logistic regression was used. The progression rates with respect to age were fitted using a local polynomial model (LOESS). In all cases, p-values were reported rounding to three digits of significance, and *P* < 0.05 was considered statistically significant.

## Results

### Patient characteristics and genetic testing results

In all, 290 Chinese patients diagnosed with OI were enrolled in the current study, where 39 (13.4%), 67 (23.1%), 162 (55.9%), and 22 (7.6%) were classified as types I, III, IV and V, respectively (Table 1). No type II was present in the current cohort. There were slightly more male patients (56.6%) than female (43.4%), and the gender ratio appeared consistent among the clinical subtypes (χ^2^ *p* = 0.93) (Table 1). The average age at the last follow-up was 12.6 ± 8.1 (standard deviation) years. Type I patients were the youngest (10.3 ± 10.3 years), followed by types IV (11.5 years ± 6.8), V (11.8 ± 6.5 years), and III (17.2 ± 8.5 years). Type III patients were significantly older than all other groups (*p* < 0.001) (types I, IV and V), among whom no significant variation was detected (*p* = 0.609).

**Table 1:** Patient characteristics

	Extended clinical subtypes				Row summary	*P* value
	I	III	IV	V		
No. of patients (%)	39 (13.4)	67 (23.1)	162 (55.9)	22 (7.6)	n = 290	
*Gender*						0.262
Female	15	29	73	9	n = 126 (43.4%)	
Male	24	38	89	13	n = 164 (56.6%)	
*Age*						
Average last-visit age (years)^a^	10.3 ± 10.3	17.2 ± 8.5	11.5 ± 6.8	11.8 ± 6.5	12.6 ± 8.1	< 0.001
*Genotypes—autosomal dominant and compound*^*b*^						
COL1A1 (I–IV) (qual v. quant^c^)	15	19	42	0	n = 76 (34.4%)	
	(2 v. 13)	(13 v. 6)	(26 v.16)			
COL1A2 (I–IV) (qual v. quant^c^)	10 (7 v. 3)	9 (9 v. 0)	50 (40 v. 10)	0	n = 69 (31.2%)	
COL1A1, COL1A2 (cmpd)	0	1	0	0	n = 1 (0.5%)	
IFITM5 (V)	0	0	0	18	n = 18 (8.1%)	
IFITM5, COL1A1(cmpd)	0	0	0	2	n = 2 (0.9%)	
*Genotypes—autosomal recessive and compound*^*b*^						
WNT1 (XV)	1	8	11	0	n = 20 (9.0%)	
SERPINF1 (VI)	0	3	6	0	n = 9 (4.1%)	
FKBP10 (XI)	0	1	5	0	n = 6 (2.7%)	
P3H1 (VIII)	0	1	1	0	n = 2 (0.9%)	
BMP1 (XIII)	0	1	0	0	n = 1 (0.5%)	
SERPINH1 (X)	0	0	1	0	n = 1 (0.5%)	
COL1A1, COL1A2, BMP1 (cmpd)	0	0	1	0	n = 1 (0.5%)	
FKBP10, COL1A1	0	1	0	0	n = 1 (0.5%)	
SEC24D, COL1A1 (cmpd)	0	0	1	0	n = 1 (0.5%)	
*Genotypes—otherwise*						
No mutation^d^	2	2	9	0	n = 13 (5.9%)	
Not tested (%)	11 (15.9)	21 (30.4)	35 (50.7)	2 (2.9)	n = 69	

^a^Plus–minus values are means ± SD. Current age: age (in years) at study cut-off date. Last-visit age: age (in years) at last hospital visits when X-rays of the spine were also taken. *IQR* inter-quartile range

^b^Roman numbers in brackets indicate OMIM subtypes. *Cmpd:* compound

^c^Qualitative mutations versus quantitative mutations

^d^ No mutation on the 18 OI risk genes tested in the current study

Among the 221 patients with genetic testing, ~ 2/3 (145 patients) carried pathogenic mutations in genes encoding type I collagen (*COL1A1*: 34.4% and *COL1A2*: 31.2%). Most of these patients were symptomatically mild or moderate, with only 16.5% (28/145) being classified as type III, lower than the cohort-wide percentage (23.1%). Single-point mutation on 5’-UTR of *IFITM5* (c.-14C > T) occurred in 20 patients, two of whom also carried pathogenic mutations in *COL1A1* (Table 1). All mutations in *COL1A1* and *COL1A2* were heterozygous. Altogether, autosomal dominant (AD) inheritance, including *COL1A1/2* and *IFITM5* variants, accounted for 75.1% (166/221) of the patients with genetic tests. Another two patients were clinically classified as type V OI without genetic testing, as they carried typical radiographic features, including hyperplastic callus, inter-osseous ossification and radial head dislocation [21]. Forty-two patients (19.0%) carried mutations in autosomal recessive (AR) genes, including *WNT1* (n = 20; 9.0%); *SERPINF1* (n = 9; 4.1%), *FKBP10* (n = 6; 2.7%) and other genes (*P3H1*, *BMP1* and *SERPINH1*) that affected only 1 ~ 2 patients. Fifteen (35.7%) of these AR patients were type III, which was over twice as high (χ^2^ *p* = 0.005) as the percentage of type III in the 166 AD patients (17.5%). This is consistent with previous reports that AR patients tend to have more severe phenotypes than AD patients [22, 23]. No mutation in OI causative genes was detected in 13 patients (5.9%) clinically diagnosed as OI. Among the 69 patients without genetic testing, their Sillence subtype distribution, with 15.9%, 30.4%, 50.7% and 2.9% in types I, III, IV and V, respectively, was comparable to the whole cohort (Table 1).

### Spine radiographic follow-ups and scoliosis prevalence

In all, 666 sitting or standing radiographs were retrieved for the 290 patients, with an average of 2.3 follow-ups (ranging 1–10) per patient. Over half (59.7%) of the patients had two follow-ups or more (Table 2), who also had a mean follow-up period of 2.5 ± 1.5 years. The patients were further stratified into non-scoliotic, mild, moderate and severe groups, based on the maximum longitudinal Cobb angle (“Methods” section). Over 70.7% (n = 205) of the patients had scoliosis (Cobb > 10°), among whom 106, 40 and 59 had mild, moderate and severe scoliosis, respectively. Only 29.3% (n = 85) were non-scoliotic (Table 2). Depending on the evidence for onset age (“Methods” section), we classified the patients with scoliosis into early-onset (EOS, n = 82), late-onset (LOS, n = 15), or ‘unknown’ (n = 108) (Table 2). Thirteen of the 82 (26.9%) EOS patients were subtype III OI, while only 1 of the 15 (6.7%) LOS and 49 of the 108 (45.3%) ‘unknown’ group were subtype III (Table 2). The onset-age groups had different last-visit ages, with EOS being the youngest (7.9 ± 2.7 years), followed by non-scoliosis (9.3 ± 8.6 years), LOS (14.6 ± 2.4 years), and ‘unknown’ (18.8 ± 7.0 years). Twenty patients of the 82 EOS had scoliosis before the age of 5, and 4 patients had it before 3 (Table 2). The onset age was related to genotypes too, with EOS accounting for 23–29% of patients in the *COL1A1/2* and untested groups, and 33–50% in the *IFITM5*, *WNT1*, *SERPINF1*, and *FKBP10* groups (Additional file 1: Table S1).

**Table 2:** Scoliosis in OI with respect to clinical subtypes

	Extended clinical subtypes				Row summary (% ^a^)	*p* value
	I	III	IV	V		
*Num of spine radiographic follow-ups*						0.19
Only once	16	27	64	10	n = 117 (40.3)	
Twice	12	12	45	4	n = 73 (25.2)	
Three times	7	6	25	5	n = 43 (14.8)	
Four times	1	12	19	1	n = 34 (11.7)	
More than four times	2	10	9	2	n = 23 (7.9)	
*Scoliosis severity (the max Cobb angle among all follow-ups)*						< 0.001
Non-scoliotic (Cobb < 10°)	26	4	50	5	n = 85 (29.3)	
Mild (Cobb 10°–25°)	13	11	74	8	n = 106 (36.5)	
Moderate (Cobb 25°–50°)	0	15	22	3	n = 40 (13.8)	
Severe (Cobb > 50°)	0	37	16	6	n = 59 (20.3)	
*Age of first scoliotic radiographs*^*b*^						
Median age (years)	7.6 ± 4.2	16.1 ± 8.3	11.6 ± 6.6	11.9 ± 6.5	12.7 ± 7.4	
*Onset age-group*^*b*^						< 0.001
EOS (< 10 years)^c^	10	13	50	9	n = 82 (28.3)	
Before 5	5	4	12	1	n = 22	
Before 3	1	1	2	0	n = 4	
LOS (≥ 10 years)^d^	1	1	13	0	n = 15 (5.2)	
Unknown^e^	2	49	49	8	n = 108 (37.2)	

^a^Out of 290 patients

^b^Only among the 205 patients with scoliosis. Age of first scoliotic radiographs is not the onset age, which usually precedes the latter to an unknown extent. Plus–minus values are means ± SD

^c^*EOS* early onset scoliosis

^d^*LOS* late onset scoliosis

^e^All cases other than early or late onset ones. The unknown group may contain both early and late onset patients

To understand the natural history of scoliosis, we analyzed the correlation of scoliosis severity with age (of maximum Cobb angles), curve property and gender. The severity was positively associated with age (one-way ANOVA *p* < 0.001), with mean ages of 8.4 ± 8.7, 10.6 ± 6.4, 15 ± 7.5, and 17.9 ± 6.3 years for the non-scoliotic, mild, moderate and severe groups, respectively (Table 3). This was consistent with the scoliosis in OI being a progressive condition. We also stratified the data-points into 5-year age-groups, and found that the severe group considerably expanded while the non-scoliotic group shrank, after the age of 25 (Additional file 1: Figure S1).

**Table 3:** Scoliosis severity with respect to genotypes

	Scoliosis severity				Row summary	*P* value
	Non-scoliotic	Mild	Moderate	Severe		
No. of patients	85	106	40	59	n = 290	
*Gender*						0.262
Female	38	39	18	31	n = 126	
Male	47	67	22	28	n = 164	
Max Cobb angles (°)	0.6 ± 2.2	15.2 ± 3.7	34.8 ± 7.5	82.3 ± 22.9	27.3 ± 31.7	< 0.001
Mean age at Max Cobb (years)	8.4 ± 8.7	10.6 ± 6.4	15 ± 7.8	17.9 ± 6.3	12.0 ± 8.1	< 0.001
*Sites of scoliosis apex of the major curve*						0.068
T1–T2	0	0	0	0	n = 0	
T3–T4	0	0	1	2	n = 3	
T5–T6	0	9	4	7	n = 20	
T7–T8	0	16	10	20	n = 46	
T9–T10	0	25	6	11	n = 42	
T11–T12	0	26	6	10	n = 42	
L1–L2	0	29	11	9	n = 49	
L3–L4	0	1	2	0	n = 3	
L5	0	0	0	0	n = 0	
*Number of curves per patient, among the 205 patients with scoliosis*						< 0.001
1 curve	0	102	30	37	n = 169	
2 curves	0	4	10	21	n = 35	
3 curves	0	0	0	1	n = 1	
*Autosomal dominant (AD)*						0.422^a^
COL1A1	23	29	11	13	n = 76	
*(Qualitative)*	*(10)*	*(15)*	*(7)*	*(9)*	*(n* = *41)*	*0.438*^b^
*(Quantitative)*	*(13)*	*(14)*	*(4)*	*(4)*	*(n* = *35)*	
COL1A2	27	27	8	7	n = 69	
*(Qualitative)*	*(22)*	*(19)*	*(8)*	*(7)*	*(n* = *56)*	*0.136*^c^
*(Quantitative)*	*(5)*	*(8)*	*(0)*	*(0)*	*(n* = *13)*	
IFITM5	3	7	3	5	n = 18	
(All singleton AD)	(53)	(63)	(22)	(25)	(n = 163)	
*AD-compound*						
COL1A1, COL1A2	0	0	0	1	n = 1	
IFITM5, COL1A1	0	1	0	1	n = 2	
*Autosomal recessive (AR) or AR/AD-compound*						
WNT1	5	5	4	6	n = 20	
SERPINF1	1	4	2	2	n = 9	
FKBP10	0	3	1	2	n = 6	
P3H1	0	1	0	1	n = 2	
BMP1	0	0	0	1	n = 1	
SERPINH1	1	0	0	0	n = 1	
(All singleton AR)	(7)	(13)	(7)	(12)	(n = 39)	
*AR/AD-compound*						
COL1A1, COL1A2, BMP1	0	0	0	1	n = 1	
FKBP10, COL1A1	0	0	0	1	n = 1	
SEC24D, COL1A1	1	0	0	0	n = 1	
No mutation^d^	3	4	3	3	n = 13	
Not tested	21	25	8	15	n = 69	

^a^Between severity grades and the three AD genes

^b^Between severity grades and the mutation types (quantitative or qualitative), within the COL1A1 patients

^c^Between severity grades and the mutation types (quantitative or qualitative), within the COL1A2 patients

^d^No mutation on the 18 OI risk genes tested in the current study. Plus–minus values are means ± SD

Interestingly, the sites of the scoliosis apex, the vertebrae corresponding to the maximum convexities, appeared borderline different among the different severity grades (*p* = 0.068). For the mild and moderate cases, the apex tended to be in the upper lumbar (L1–L2) or lower thoracic (T7–T12) regions, whereas for the severe cases, the apex was most commonly found in the T7–T8 region (Table 3). Among the 205 patients with scoliosis, 169 (82.4%), 35 (17.1%) and 1 (0.5%) patient(s) developed single, double and triple curves, respectively. The number of curves was positively correlated with the scoliosis severity (χ^2^ *p* < 0.001) (Table 3). On the other hand, gender did not show any correlation with severity grades (χ^2^ *p* = 0.262).

### Scoliosis severity with respect to genetic risk factors

Patients with pathogenic variants in *COL1A1* or *COL1A2* alone tended to have a milder form of scoliosis. In particular, among the 76 patients carrying *COL1A1* mutations, 23 were non-scoliotic and 29 were mildly scoliotic, accounting for > 2/3 of *COL1A1* patients. Only 11 (14.5%) and 13 (17.1%) patients in this group had moderate and severe scoliosis, respectively. Similarly, patients with mutations in *COL1A2* also tended to be milder. Fifty-four of the 69 (78.3%) *COL1A2* patients were non-scoliotic or mildly scoliotic, while only 8 and 7 had moderate and severe scoliosis, respectively. Patients carrying qualitative mutations (i.e. missense mutations) were known to have more severe phenotypes than those carrying quantitative ones (frameshift or stop codon) [24]. In moderate and severe cases, the proportion of qualitative mutations in* COL1A1 *and* COL1A2 *is higher than quantitative mutations. In both *COL1A1* (χ^2^ *p* = 0.438) and *COL1A2* (χ^2^ *p* = 0.136), carrying qualitative or quantitative mutations did not show a significant difference in scoliosis severities (Table 3).

For the 20 patients with *IFITM5* mutation, scoliosis severities were not more biased towards the non-scoliotic or mild groups. Three (15%) patients were non-scoliotic (Table 3) and ten (50%) were mild or moderate. Patients with mutations in AR genes, including *WNT1*, *SERPINF1*, *FKBP10*, *P3H1* and *BMP1*, had similar severity distributions as *IFITM5* (Table 3). Among these AR patients, seven (17.9%) were non-scoliotic, while 13 (33.3%), 7 (17.9%), and 12 (30.8%) had mild, moderate and severe scoliosis, respectively. There was no difference between the severity of patients with *IFITM5* mutation and those with mutations in *WNT1*, the most common AR gene (χ^2^ *p* = 0.81), or the patients with other AR gene mutations (χ^2^ *p* = 0.95). In particular, all six patients with mutations in *FKBP10* (type XI OI) had scoliosis [25]. Due to the small number of patients (n = 3) with AD/AR compound mutations, no statistics was performed (Table 3).

Interestingly, among the 13 patients without mutations in the tested genes, scoliosis severities were also not biased towards non-scoliotic or mild grades. On the other hand, the severity distribution among the 69 untested patients was highly similar to that of the 221 tested patients (χ^2^ *p* = 0.93), suggesting the representation of the cohort and the accuracy of clinical diagnoses based on radiographic features.

### Scoliosis severity with respect to non-genetic factors

Skeletal maturity, including the Risser sign and the closure age of triradiate cartilage, was considered to be related to the curve acceleration phase in adolescent idiopathic scoliosis (AIS) [26]. To reveal the relation between OI scoliosis and skeletal maturity, we graded both the Risser sign and the triradiate cartilage closure from the patients’ radiographs.

Due to the relatively young ages in our cohort, 179 patients (61.7%) still had open triradiate cartilage during their last radiographs. Another 50 patients were over the age of 18 and their triradiate cartilage were considered closed with the closure age unknown. This left 61 patients whose closure age were captured by radiographs (Table 4). Among them, the closure age most commonly occurred at the ages of 13 to 15 (Table 4). Similarly, the majority of patients (n = 169, 58.3%) had Risser sign of grade 0 in their last radiographs, with another 20 (6.9%), 34 (11.7%), and 77 (26.5%) patients having Risser signs of grades 1–2, 3–4, and 5, respectively (Table 4).

**Table 4:** Scoliosis severity with respect to non-genetic factors, including clinical phenotypes and treatment history

Non-genetic factors	Scoliosis severity				Row summary	*p* value
	Non-scoliotic	Mild	Moderate	Severe		
No. of patients	85	106	40	59	n = 290	
*Age of triradiate cartilage closure*						
Still open	67	80	17	15	n = 179	
Closed	10	18	14	19	n = 61	
Closed at 11 years	0	1	0	0	n = 1	
Closed at 12 years	1	0	1	2	n = 4	
Closed at 13 years	4	5	3	2	n = 14	
Closed at 14 years	2	5	4	1	n = 12	
Closed at 15 years	2	2	4	7	n = 15	
Closed at 16 years	0	3	1	3	n = 7	
Closed at 17 years	0	2	1	3	n = 6	
Closed at 18 years	1	0	0	1	n = 2	
Closed and overaged (> 18 years)	8	8	9	25	n = 50	
*Risser sign skeletal maturity age (based on last radiograph follow-up)*						< 0.001
Grade = 0	68	76	14	11	n = 169	
Grade = 1–2	4	6	4	6	n = 20	
Grade = 3–4	6	11	6	11	n = 34	
Grade = 5	11	15	15	36	n = 77	
*Lower-limb discrepancy (LLD)*						
No. of patients with LLD	28	54	18	40	n = 140	< 0.001
(%with LLD)	(32.9%)	(50.9%)	(45.0%)	(67.8%)	(48.3%)	
*BMD Z-scores on last follow-ups*						
Average BMD Z-scores^b^	− 1.8 ± 2.0	− 1.8 ± 2.2	− 2.6 ± 2.1	− 3.5 ± 1.9	− 2.2 ± 2.2	< 0.001
*Surgical interventions of the spine*						
Never treated^a^	85	106	40	34	265 (91.4%)	
SZH	0	0	0	22	22 (7.6%)	
Extramural	0	0	0	3	3 (1.0%)	
*BMD improvement drugs used*						0.011
Zoledronate	59	68	22	21	n = 170	
Pamidronate	20	29	11	24	n = 84	
Ibandronate	0	1	1	0	n = 2	
Alendronate	0	1	0	0	n = 1	
Denosumab	0	0	0	1	n = 1	
Never treated^c^	6	7	6	13	n = 32	
Average age of first drug use^d^	6 ± 7.6	6.3 ± 5.1	8.1 ± 4.8	10.3 ± 6.0	7.2 ± 6.2	

^a^Never treated with spine surgeries

^b^Based on 266 patients who had at least one BMD examination

^c^Never treated with bisphosphonates or monoclonal antibodies

^d^Excluding the 32 patients who never received drug treatment. Plus–minus values are means ± SD

We next asked whether scoliosis was affected by lower-limb deformities. Almost half (n = 140, 48.3%) of the cohort had lower-limb discrepancies (LLD), and its prevalence was significantly associated with scoliosis severity (χ^2^ *p* < 0.001). The percentage of patients with LLD was significantly higher in the severe group (67.8%) than the non-scoliotic (32.9%), mild (50.9%), and moderate (45.0%) groups (Table 4).

In patients with AIS, BMD was known to be lower, although no correlation was found with the severity of spinal deformities [27]. Among the 266 patients with BMD measurements, we found their age- and gender-adjusted scores (Z-scores) were generally below normal (− 2.2 ± 2.2). We also found that the Z-scores were significantly correlated with scoliosis severities (*p* < 0.001). Post-hoc analysis by Tukey honest significant difference test showed that the severe group had the lowest BMD Z-scores (− 3.5 ± 1.9), as compared with the non-scoliotic (*p* < 0.001) and mild (*p* < 0.001) groups. No difference (*p* = 0.99) was observed between the non-scoliotic and the mild groups.

Drug use may improve BMD and change the course of the scoliosis development in OI. Over 90% of the patients were never treated for surgical interventions on their spine (Table 4). BMD improvement drugs, most dominantly bisphosphonates, were used at least once in 89% of the patients (n = 258). Zoledronate injection was the most dominant (58.6%), followed by pamidronate (29.0%). The use of other bisphosphonates or monoclonal antibodies was only reported in 4 patients. Thirty-two patients never received any BMD improvement drugs, and 13 patients had severe scoliosis (Table 4). We found that the age of first drug use also predicted scoliosis severity (*p* < 0.001), with the severe group having evidently higher age of first drug use (average 10.3yo) than other groups (on average 6, 6.3 and 8.1 for the non-scoliotic, mild, and moderate groups, respectively) (Table 4).

### Multi-variate analyses of progression into advanced stages of scoliosis

Next, we focused on OI scoliosis severity with respect to individual genetic and non-genetic factors. In reality, these factors are often interdependent. For example, skeletal maturity measures, including the Risser sign and the closure age of triradiate cartilage, are highly dependent on the patient ages of available radiographs. Sillence grades are dependent on the presence of lower-limb discrepancy and scoliosis, which in turn depends on genetics. BMD Z-scores depend on genetics and drug history. Thus, genetics (including affected genes, inheritance pattern and mutation types), age, drug history (including drug types and ages of first use) and gender are “fundamental” independent factors, whereas Sillence grades, the presence of LLD, and skeletal maturity may be considered “intermediate” factors that are themselves the outcomes of one or multiple other fundamental factors. We asked how some of these factors may increase or decrease the chance (in terms of odds ratio) of progressing into the advanced stages scoliosis (moderate or severe) of scoliosis.

We considered a multi-variate logistic regression model, whereby the radiographs were binarized into: (1) non-scoliotic or mild, and; (2) advanced stages of scoliosis (moderate or severe). As Sillence grading had taken scoliosis into consideration, we excluded Sillence grading from the model, and considered age corresponding to the maximum Cobb angles, gender, genotypes, drug history, BMD Z-scores and LLD as predictors. Regression results showed that age was the most significant predictor (Table 5), with each year older contributing to an increased odds ratio (OR) of 1.13 (95% CI 1.07–1.2, *p* < 0.001) of developing moderate or severe scoliosis. Gender, on the other hand, was not predictive of progression (Table 5).

**Table 5:** Logistic regression model revealed key predictors of scoliosis severity in OI

	OR (95% CI)	*p* values
*Dataset 1: all 290 patients*		
Age (per year)	1.13 (1.07 ~ 1.2)	*p* < 0.001***
Gender female (vs. male)	1.43 (0.78–2.63)	0.25
Genotypes		
COL1A1 or COL1A2	Reference	–
IFITM5	3.71 (1.13–12.25)	0.029*
WNT1	3.37 (1.06–10.69)	0.037*
All other AR genes	3.16 (1.03–9.63)	0.041*
No mutation	1.92 (0.43–7.91)	0.37
Not tested	1.33 (0.6–2.88)	0.473
Drugs used		
Never^a^	Reference	–
Pamidronate	3.78 (1.09–13.94)	0.04*
Zoledronate	1.96 (0.59–7)	0.285
BMD Z-scores (per SD)	0.82 (0.7–0.95)	0.011*
Has LLD (vs. no LLD)	1.07 (0.57–1.99)	0.825
*Dataset 2: the 145 COL1A1/2 patients only*		
Age (per year)	1.13 (1.05–1.22)	0.001**
Gender female (vs. male)	1.81 (0.73–4.55)	0.199
Genotype COL1A2^b^	0.28 (0.1–0.71)	0.01*
Mutation-type qualitative^c^	3.84 (1.34–12.52)	0.017*
BMD Z-scores (per SD)	0.79 (0.61–0.99)	0.048*
Has LLD (vs. no LLD)	0.57 (0.21–1.47)	0.253

*OR* odds ratio, *CI* confidence interval, *SD* standard deviation, *LLD* lower-limb discrepancy

^a^Never treated with bisphosphonates or monoclonal antibodies

^b^Reference is COL1A1

^c^Reference is “Quantitative”. Model: hasAdvancedScoliosis ~ covariates. Advanced scoliosis: moderate or severe scoliosis. Covariates for dataset 1 included age, gender, genotypes, drugs used, BMD (Z scores) and LLD. Covariates for dataset 2 included age, gender, genotypes, mutation types, BMD (Z scores) and LLD

*p < 0.05; **p < 0.01; ***p < 0.001

Genetic variants appeared predictable for moderate/severe and severe scoliosis (Table 5). In particular, *IFITM5* and *WNT1* mutations increased the OR by 3.71 (95% CI 1.13–12.25, *p* = 0.029) and 3.37 (95% CI 1.06–10.69, *p* = 0.037) times, respectively. Mutations in all other AR genes also significantly increased the OR by 3.16 (95% CI 1.03–9.63, *p* = 0.041) times.

Drug history seems to be relevant to progression too. The use of pamidronates increased the OR by 3.78 times (95% CI 1.09–13.94, *p* = 0.04). But we cautioned the interpretation of this result, as pamidronate is an older bisphosphonate and was used by older patients, making it a confounder of the age factor. Bone mineral density (BMD) was a strong predictor, with each one standard deviation increase resulting in 0.82 (95% CI 0.7–0.95, *p* = 0.011) times lower OR. The lower-limb discrepancy did not appear to affect the severity grades at all (Table 5).

Since patients with *COL1A1* and *COL1A2* variants together constituted about 2/3 of the cohort, we asked if scoliosis severity was differentially affected by the two genes. We applied the same three models above (excluding drug history and adding mutation types), but to the 145 patients carrying mutations in these two genes only. We found that *COL1A2* appeared significantly less severe than *COL1A1*, with an OR of 0.28 (95% CI 0.1–0.71, *p* = 0.01) for progression into moderate or severe scoliosis (Table 5). In terms mutation types, qualitative mutations in *COL1A1/2* were overall more damaging than quantitative variants, with an OR of 3.84 (CI 1.34–12.52, *p* = 0.017).

### Progression rate with respect to genetic and non-genetic factors

**Fig. 1:**
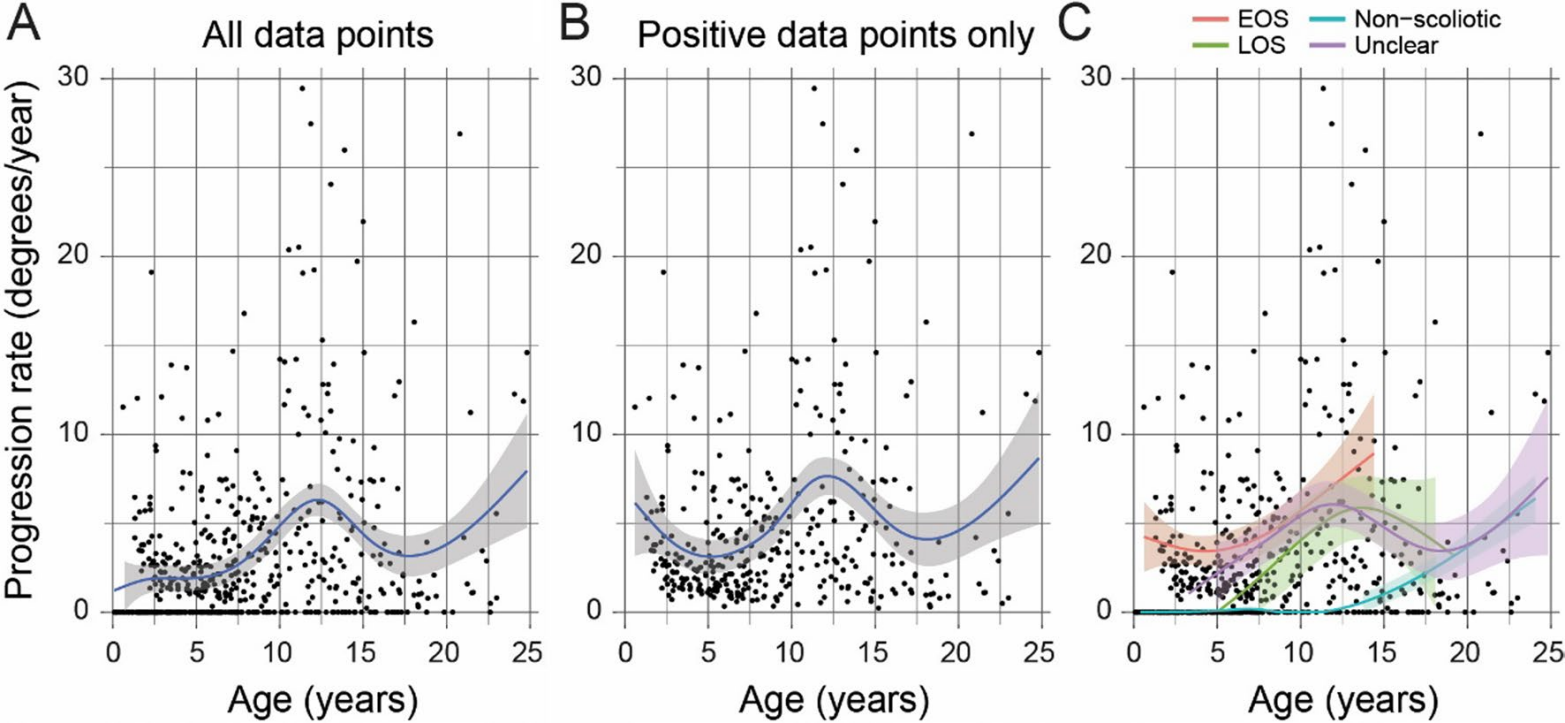
Progression rate estimates with respect to age. Each data point is an estimate of the progression rate for two adjacent radiographs of a patient. The age is the mid-point of the ages associated with those two radiographs. The curves were fitted results of LOESS regressions, and the shaded areas were the corresponding standard error envelopes. **A** All data points of progression rate estimates, including estimates for patients without scoliosis (many were zero-valued), were used. **B** Only positive data points were used. **C** The data points were stratified according the onset age groups. *EOS* early onset, *LOS* late onset, *‘unknown’* all other patients with scoliosis

We then examined the impact of risk factors on the progression rate of scoliosis. We only considered the preoperative Cobb angles of major curves and calculated the progression slope (“Methods” section). We also included patients without scoliosis at the cutoff date of this study. Overall, the mean progression rate was 2.7°/year (95% CI 2.4–3.0) with a peak at ~ 12.5 years of age (Fig. 1A). If we focused on the positive progression rates only (excluding most non-scoliotic cases), we found that apart from a peak at ~ 12.5 years there is also a peak before the age of 5 (Fig. 1B). We stratified our patients according to their onset age-groups, and found that the EOS and ‘unknown’ group both had a peak before 5, while the LOS had one around 14 years, and the non-scoliotic had a monotonically increasing curve (Fig. 1C). We performed a simple multivariate regression between progression rates and covariates, which showed that with COL1A2 having a lower progression rate (1.8°/year, 95% CI 0.9–2.6, *p* = 0.015) than the baseline level (represented by *COL1A1*), and patients with *WNT1* mutations had higher progression rates on average (4.1°/year, 95% CI 2.9–5.2, *p* = 0.028) (Additional file 1).

### Surgical interventions in some patients with severe scoliosis

In all, 25 patients (all were severely scoliotic) underwent spinal surgeries, which represent 42.8% of the 59 patients with severe scoliosis. These patients had an average preoperative Cobb angle of 85.3° (IQR 64–96). After spinal surgery, they enjoyed an average reduction of 33° (IQR 23–40). These patients are currently being followed up. The surgical details and follow-up outcomes will be reported separately.

## Discussion

Scoliosis is one of the most prevalent conditions among osteogenesis imperfecta patients and is well-known to be progressive with age [11, 12]. It has a huge impact on the quality of life among OI patients, yet both its disease causes and courses, which are vastly different from other more common forms of scoliosis, remain poorly understood. Previous studies of OI scoliosis either had small sample sizes [16] or covered incomplete genotypes [12]. In this study, we reported a retrospective study of OI scoliosis outcomes and progression based on a large cohort of 290 OI patients, of whom up to 76% had confirmed genetic information.

We stratified the cohort by four scoliosis outcome grades, including non-scoliotic, mild, moderate and severe, as measured by their maximum (preoperative, if any) Cobb angles of the major curves. We then performed univariate and multivariate analyses between the outcome and a set of genetic and non-genetic factors. We found that patients with *COL1A1* and *COL1A2* genotypes were strongly biased towards having mild or no scoliosis at all, whereas patients with pathogenic variants on *IFITM5*, *WNT1* and other recessive genes did not display such a pattern. Due to the relatively small number of cases in *IFITM5* and recessive genes, it was difficult to statistically delineate their effects on the outcomes, although their fractions of moderate or severe cases were comparable.

Within the two collagen genes, *COL1A2* was less damaging than *COL1A1* in progressing into advanced stages of scoliosis. The mutation types, in terms of qualitative or quantitative changes, had a weak influence on the outcomes. Neither within each collagen gene nor the two genes combined did the mutation types have a significant association with the severity grades. Among the non-genetic factors, we found that skeletal maturity, lower-limb deformities, and drug history were all individually associated with severity outcomes (Table 4), although when taken together into a multivariate logistic regression model, many of them (including drug history and lower-limb deformities) had weak or no associations (Table 5). A likely explanation is that OI scoliosis is highly age-dependent, thus the contributions of many age-dependent factors, such as skeletal maturity and LLDs, were largely absorbed by the age variable itself. Drug history may reflect patient age too, as older patients were more likely to either take pamidronate or even did not take drugs at all.

We included all patients, even those considered non-scoliotic at the cutoff date, for estimating the progression rates, which were estimated by dividing the angle difference by the age difference between successive datapoints of the same individuals. In fact, 40 of the 85 patients without scoliosis had multiple follow-ups, all of which had Cobb angles < 10°. All datapoints from these patients were included, to ensure that the progression rate estimation was unbiased and accurate. We estimated an overall progression rate of 2.7°/year, which was highest among adolescents and young adult age-groups (10–20 years), and was lower in *COL1A2* than in *COL1A1*.

Overall, we noted both the outcome grades and progression rates were more severe in our cohort than in the literature. At 70.7%, the prevalence of scoliosis was considerably higher in our cohort than previously reported [11, 12, 16]. The overall progression rate of 2.7°/year was in close range to but also slightly higher than previously reported estimates of 2.3–2.6°/year [11, 12]. Both of these could be due to the inclusion of non-*COL1A1/2* patients in our study and socioeconomic reasons. Unlike in the West where 85–90% of the OI patients seeking medical treatments were affected by *COL1A1/2* mutations [28, 29], our cohort and others in China consistently included 2/3–3/4 of such patients only [30]. Subtype I, the clinically mild form of OI, only made up 13.4% of our cohort, whereas they usually accounted for ~ 40% in western cohorts [11, 12, 16, 31]. At an average age of 12.0 years corresponding to the maximum Cobb angle, our cohort is also older than the study that reports 54% prevalence at a mean age of 7 years [11].

We also studied the onset age of scoliosis in OI. Based on radiographic evidence, we stratified the scoliosis group into early-onset (EOS), late-onset (LOS) and ‘unknown’. The ‘unknown’ group represented over half of all scoliotic OI (108 out of 205, Table 2), and was thus labelled because of the missing information regarding their spinal condition before 10. We found that there are > 5 times more EOS than LOS in our cohort. Overall, the ‘unknown’ group, the exact onset age among whom cannot be confirmed, also behaved quite similarly to the EOS (Fig. 1C). In fact, there were more patients who were diagnosed with scoliosis before the age of 5 (n = 22) than after 10 (n = 15) (Table 2). We postulated that per the current consensus of cutoff age of 10 for EOS/LOS, majority of the scoliosis OI patients seeking treatment in our hospital may fall into the EOS category. Our results suggest it is important to monitor the spinal health of these patients, even though most of their medical interventions currently focus on the limbs and bone densities.

We are aware that multiple other factors may limit the accuracy in our study. Manual reading of Cobb angles and nonstandard radiographic positioning may add noise the data. We found that 52 of the 606 preoperative Cobb data-points were smaller than their immediate previous follow-ups, with an average reduction of 5.6 ± 3.7 degrees among them. Upon reexamining the radiographs, we confirmed that all of these were real, and that all but one were posture-induced. Since 48% of our cohort had LLD and other lower-limb conditions were common, standard upright radiographic postures were difficult to attain for many patients, which in turn caused considerable difficulties in accurately reading Cobb angles, even for experienced physicians. There was inevitably a certain amount of data noise attributable to such cause. The only other case involved a girl who experienced a Cobb angle drop of 18° over the course of 2 years. Re-examining the records showed that the girl had been wearing bracing for 2 years, after which the Cobb angle appeared reduced and stabilized (Additional file 1: Figure S2). Bracing has proven positive effects on other common forms of scoliosis during adolescence [32], but its use in OI has been disputed. Early studies suggested bracing was not effective in OI scoliosis [33, 34], and as such it was not used often in our cohort (< 10 patients) and other recent studies [11]. As our case showed and as noted in [11], with the use of modern anti-osteoporotic agents such as bisphosphonates, a second look into the effects of orthosis in OI scoliosis is needed in future studies.

Although we tried to make our cohort as representative as possible by including all consecutive cases, the non-*COL1A1/2* patients still only represented a minority (~ 1/3). Since eight genes were involved among these cases, the number of cases per genotype was rather small. This distribution bias may cause difficulty in estimating genotype-specific effects on OI scoliosis. We addressed this by a two-step approach, whereby the two collagen genes were first considered as a single group, before a second analysis on the 145 patients affected by these two genes only was conducted, where *COL1A1* and *COL1A2* were now treated as separate groups. Sillence classification was often used as an independent variable to explain the scoliosis outcomes [11, 12], although it is well-known that scoliosis itself was part of the criteria for grading the Sillence subtypes [7]. To avoid circularity, we did not present the results of analyses using it as a covariate.

Lastly, it is noteworthy that the cases in the current study only represented OI patients seeking treatment at our hospital, as a result of which some milder cases not needing medical treatment were not screened. Our results thus may appear more severe than the actual situation among the broader OI community.

## Conclusions

In all, a comprehensive study of scoliosis in osteogenesis imperfecta was undertaken to identify the genetic and non-genetic factors affecting its severity and progression, and it is hoped insights from this study may be helpful in making certain clinical decisions.

### Supplementary Information

**Additional file 1**. Supplementary information.

**Additional file 2**. Anonymized genetic testing results of the 54 patients newly reported in the current study.

## Data Availability

Part of the scripts for analyzing the data is deposited on github: https://github.com/HKUSZH/OI-scoliosis. Due to privacy concerns, clinical data used in this study will not be made publicly available. However, anonymized information may be obtained upon reasonable request to the authors.

## References

[1] Forlino A, Marini JC (2016). Osteogenesis imperfecta. Lancet.

[2] Chen P (2022). Phenotypic spectrum and molecular basis in a Chinese cohort of osteogenesis imperfecta with mutations in type I collagen. Front Genet.

[3] Chen P (2022). Patient-reported outcomes in a Chinese cohort of osteogenesis imperfecta unveil psycho-physical stratifications associated with clinical manifestations. Orphanet J Rare Dis.

[4] Marini JC (2017). Osteogenesis imperfecta. Nat Rev Dis Primers.

[5] Liang X (2022). Comprehensive risk assessments and anesthetic management for children with osteogenesis imperfecta: a retrospective review of 252 orthopedic procedures over 5 years. Paediatr Anaesth.

[6] Sillence DO, Rimoin DL, Danks DM (1979). Clinical variability in osteogenesis imperfecta-variable expressivity or genetic heterogeneity. Birth Defects Orig Artic Ser.

[7] Van Dijk FS, Sillence DO (2014). Osteogenesis imperfecta: clinical diagnosis, nomenclature and severity assessment. Am J Med Genet A.

[8] Cho TJ (2012). A single recurrent mutation in the 5'-UTR of IFITM5 causes osteogenesis imperfecta type V. Am J Hum Genet.

[9] Hresko MT (2013). Clinical practice. Idiopathic scoliosis in adolescents. N Engl J Med.

[10] Bronheim R (2019). Scoliosis and cardiopulmonary outcomes in osteogenesis imperfecta patients. Spine (Phila Pa 1976).

[11] Anissipour AK (2014). Behavior of scoliosis during growth in children with osteogenesis imperfecta. J Bone Joint Surg Am.

[12] Sato A (2016). Scoliosis in osteogenesis imperfecta caused by COL1A1/COL1A2 mutations—genotype-phenotype correlations and effect of bisphosphonate treatment. Bone.

[13] Cheung JPY (2018). Curve progression in adolescent idiopathic scoliosis does not match skeletal growth. Clin Orthop Relat Res.

[14] Noshchenko A (2015). Predictors of spine deformity progression in adolescent idiopathic scoliosis: a systematic review with meta-analysis. World J Orthop.

[15] Liu G (2017). The genetic implication of scoliosis in osteogenesis imperfecta: a review. J Spine Surg.

[16] Kashii M (2019). Development of scoliosis in young children with osteogenesis imperfecta undergoing intravenous bisphosphonate therapy. J Bone Miner Metab.

[17] Engelbert RH (2003). Scoliosis in children with osteogenesis imperfecta: influence of severity of disease and age of reaching motor milestones. Eur Spine J.

[18] Arponen H, Makitie O, Waltimo-Siren J (2014). Association between joint hypermobility, scoliosis, and cranial base anomalies in paediatric Osteogenesis imperfecta patients: a retrospective cross-sectional study. BMC Musculoskelet Disord.

[19] Zhu F (2021). A comparison of foot posture and walking performance in patients with mild, moderate, and severe adolescent idiopathic scoliosis. PLoS ONE.

[20] Williams BA (2014). Development and initial validation of the Classification of Early-Onset Scoliosis (C-EOS). J Bone Joint Surg Am.

[21] Glorieux FH (2000). Type V osteogenesis imperfecta: a new form of brittle bone disease. J Bone Miner Res.

[22] Rohrbach M, Giunta C (2012). Recessive osteogenesis imperfecta: clinical, radiological, and molecular findings. Am J Med Genet C Semin Med Genet.

[23] Li S (2020). Genotypic and phenotypic analysis in Chinese cohort with autosomal recessive osteogenesis imperfecta. Front Genet.

[24] Li LJ (2019). Genotype-phenotype relationship in a large cohort of osteogenesis imperfecta patients with COL1A1 mutations revealed by a new scoring system. Chin Med J (Engl).

[25] Tan Z (2023). Clinical features and molecular characterization of Chinese patients with FKBP10 variants. Mol Genet Genom Med.

[26] Nault ML (2010). A modified Risser grading system predicts the curve acceleration phase of female adolescent idiopathic scoliosis. J Bone Joint Surg Am.

[27] Cheng JC (2000). Generalized low areal and volumetric bone mineral density in adolescent idiopathic scoliosis. J Bone Miner Res.

[28] Ohata Y (2020). Correction to: Comprehensive genetic analyses using targeted next-generation sequencing and genotype-phenotype correlations in 53 Japanese patients with osteogenesis imperfecta. Osteoporos Int.

[29] Maioli M (2019). Genotype-phenotype correlation study in 364 osteogenesis imperfecta Italian patients. Eur J Hum Genet.

[30] Liu Y (2017). Gene mutation spectrum and genotype-phenotype correlation in a cohort of Chinese osteogenesis imperfecta patients revealed by targeted next generation sequencing. Osteoporos Int.

[31] Patel RM (2015). A cross-sectional multicenter study of osteogenesis imperfecta in North America—results from the linked clinical research centers. Clin Genet.

[32] Uno H, Wei LJ, Hughes M (2014). Effects of bracing in adolescents with idiopathic scoliosis. N Engl J Med.

[33] Cristofaro RL (1979). Operative treatment of spine deformity in osteogenesis imperfecta. Clin Orthop Relat Res.

[34] Yong-Hing K, MacEwen GD (1982). Scoliosis associated with osteogenesis imperfecta. J Bone Joint Surg Br.

