# Supplementary material for: Scoliosis in osteogenesis imperfecta: identifying the genetic and non-genetic factors affecting severity and progression from longitudinal data of 290 patients
Source: Orphanet J Rare Dis. 2023 Sep 20;18:295. doi: 10.1186/s13023-023-02906-z (PMC10510243; doi:10.1186/s13023-023-02906-z)
Supplement: Supplementary file 1 — Additional file 1. Supplementary information. [file 13023_2023_2906_MOESM1_ESM.docx]

# **Scoliosis in Osteogenesis Imperfecta: Identifying the Genetic and Non-genetic Factors Affecting Severity and Progression from Longitudinal Data of 290 Patients**

Peikai Chen^1,2*#^, Yapeng Zhou^1*^, Zhijia Tan^1,3*^, Yunzhi Lin^1^, Daniel Li-Liang Lin^1^, Jingwei Wu^1^, Zeluan Li^1^, Hiu Tung Shek^1^, Jianbin Wu^1^, Yong Hu^1,3^, Feng Zhu^1,3^, Danny Chan^1,2^, Kenneth Man-Chee Cheung^1,3^, Michael Kai-Tsun To^1,3#^

# **Supplementary Information**

### **Progression rate with respect to genetic and non-genetic factors**

We performed simple linear regression between progression rates and each variable, including age (five-year groups and Risser signs), gender, genetic variants and drug history. We found that both age-groups (p<0.001) and Risser signs (p<0.001) explained the progression rates, while gender did not affect it (p=0.69). Genotypes also significantly explained the progression rates (p= 0.004), with *COL1A2* having a lower progression rate (1.8 degrees/year, 95% CI 0.9 ~ 2.6, p=0.015) than the baseline level (represented by *COL1A1*). Patients with *WNT1* mutations had higher progression rates on average (4.1 degrees/year, 95% CI 2.9~5.2, p= 0.028). Other genetic variants or drug history did not affect the progression rates either. Since age and genetic mutations affected the progression rates, we further performed a multiple linear regression (dependent variable: the progression rates; independent variables: age and genetic variants). The results showed similar trends as above. The adolescent age-group (10~15 years) had the highest progression rate (3.9 degrees/year, 95% CI 3.0 ~ 4.8, p<0.001), followed by the 15~20 years group (3.4 degrees/year, 95% CI 2.3 ~ 4.6, p=0.028) (Table S2). Even combined with the age-groups, patients with *COL1A2* variants still had lower progression rates (1.1 degrees/year, 95% CI -0.4 ~ 2.6) (Figure S3A-B) while patients with *WNT1* mutations still had higher progression rates (3.6 degrees/year, 95% CI 2.5 ~ 4.8) (Table S2).

## **SUPPLEMENTARY TABLES**

### **Table S1 Progression rates with respect to age-groups and genotypes.**

|  | non-scoliotic | EOS  (%*) | LOS  (%*) | Unconfirmed | Row  Summary † |
| --- | --- | --- | --- | --- | --- |
| Num of patients | 85 | 82 | 15 | 108 | n=290 |
| Median age of first scoliotic radiographs (yrs) | N.A. | 6.6±2.1 | 13±1.9 | 17.4±7.1 |  |
| **Genotypes** | | | | |  |
| COL1A1 | 23 | 19 (25.0) | 6 (7.9) | 28 | n=76 (34.4%) |
| COL1A2 | 27 | 16 (23.2) | 5 (7.2) | 21 | n=69 (31.2%) |
| COL1A1, COL1A2 | 0 | 0 | 0 | 1 | n=1 (0.5%) |
| IFITM5 | 3 | 8 (44.4) | 0 | 7 | n=18 (8.1%) |
| IFITM5, COL1A1 | 0 | 1 (50.0) | 0 | 1 | n=2 (0.9%) |
| WNT1 | 5 | 10 (50.0) | 0 | 5 | n=20 (9.0%) |
| SERPINF1 | 1 | 3 (33.3) | 1 (11.1) | 4 | n=9 (4.1%) |
| FKBP10 | 0 | 3 (50.0) | 0 | 3 | n=6 (2.7%) |
| P3H1 | 0 | 1 (50.0) | 0 | 1 | n=2 (0.9%) |
| BMP1 | 0 | 0 | 0 | 1 | n=1 (0.5%) |
| SERPINH1 | 1 | 0 | 0 | 0 | n=1 (0.5%) |
| COL1A1, COL1A2, BMP1 | 0 | 0 | 0 | 1 | n=1 (0.5%) |
| FKBP10, COL1A1 | 0 | 0 | 0 | 1 | n=1 (0.5%) |
| SEC24D, COL1A1 | 1 | 0 | 0 | 0 | n=1 (0.5%) |
| No mutation | 3 | 1 (7.7) | 1 (7.7) | 8 | n=13 (5.9%) |
| Not tested | 21 | 20 (29.0) | 2 (2.9) | 26 | n=69 |

EOS: early onset scoliosis. LOS: late onset scoliosis. Unconfirmed: scoliotic evidence available after 10 but information of when the condition developed was unavailable. * Percentages calculated based on row-wise summations. † Percentages calculated based on 221 patients whose genetics were tested.

### **Table S2 Progression rates with respect to age-groups and genotypes.**

|  | Estimate  (degrees/year) | 95% CI | P values | |
| --- | --- | --- | --- | --- |
| (Intercept) | 2.2 | 1.4~3.0 | <0.001 |  |
| **Age groups *** | | | |  |
| 0 ~ 5 years | +0 | Reference | --- |  |
| 5 ~ 10 years | -0.08 | -1.0 ~ 0.8 | 0.859 |  |
| 10 ~ 15 years | +1.7 | 0.8 ~ 2.6 | <0.001 | *** |
| 15 ~ 20 years | +1.2 | 0.1 ~ 2.4 | 0.028 | * |
| 20 ~ 25 years | +1.2 | -0.4 ~ 2.8 | 0.127 |  |
| 25 ~ 30 years | +0.5 | -1.2 ~ 2.2 | 0.558 |  |
| 30 ~ 35 years | -1.0 | -4.1 ~ 2.2 | 0.543 |  |
| > 40 years | -1.1 | -3.8 ~ 1.6 | 0.419 |  |
| **Genotypes**† | | | |  |
| COL1A1 | +0 | Reference | --- |  |
| COL1A2 | -1.1 | -1.9 ~ -0.3 | 0.009 | ** |
| IFITM5 | +0.5 | -0.9 ~ 1.8 | 0.505 |  |
| WNT1 | +1.5 | 0.3 ~ 2.6 | 0.012 | * |
| FKBP10 | +1.0 | -1.4 ~ 3.5 | 0.417 |  |
| SERPINF1 | +0.07 | -1.8 ~ 2.0 | 0.941 |  |
| Other AR genes | +0.8 | -1.9 ~ 3.4 | 0.573 |  |
| Compound† | +1.3 | -0.8 ~ 3.4 | 0.212 |  |
| No mutations | -1.2 | -2.7 ~ 0.3 | 0.125 |  |
| Not tested | -0.05 | -0.9 ~ 0.8 | 0.899 |  |

† AD and/or AR compound mutations.

## **SUPPLEMENTARY FIGURES**


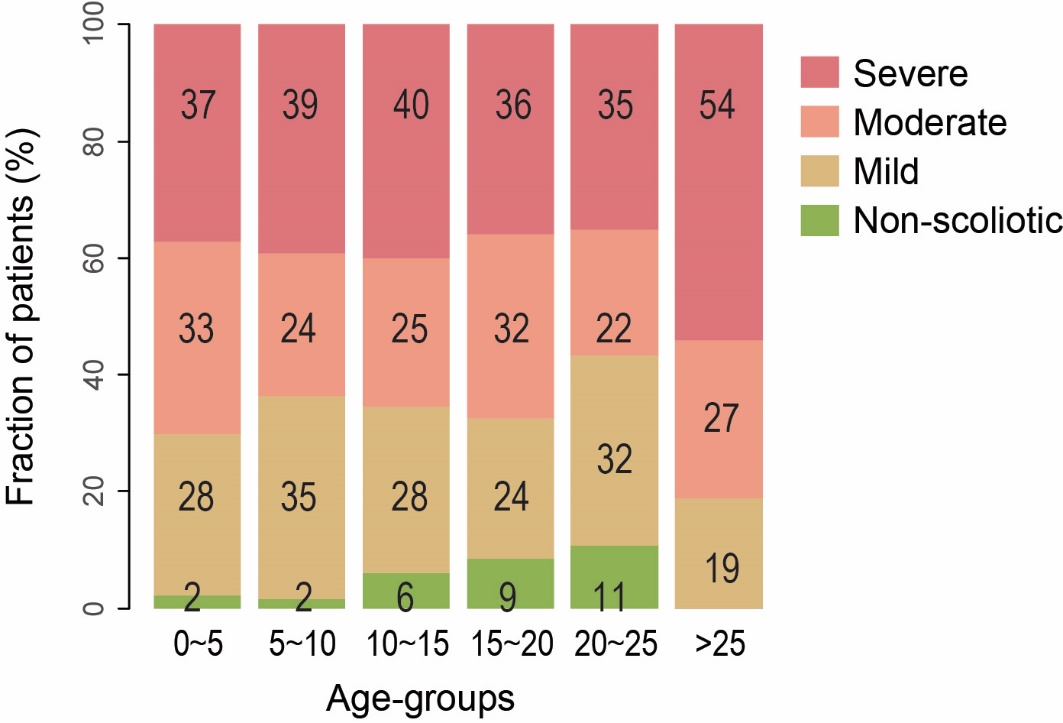


**Figure S1** Stratifying the data-points into four severity grades and six age-groups. Numbers in the bar-chart indicate percentages within each age-group.


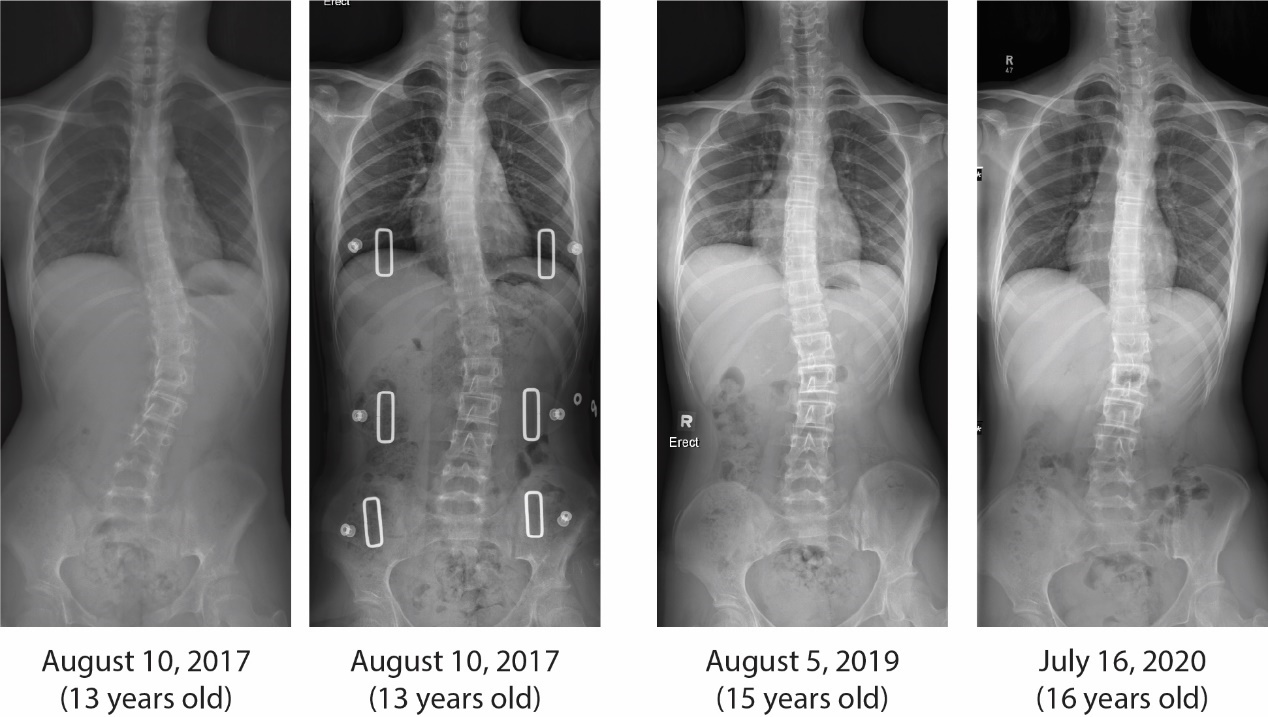


**Figure S2** A case of bracing in an adolescent girl. Left two: before and after wearing bracing in August 2017. Right two: without wearing bracing in 2019 and 2020.

**
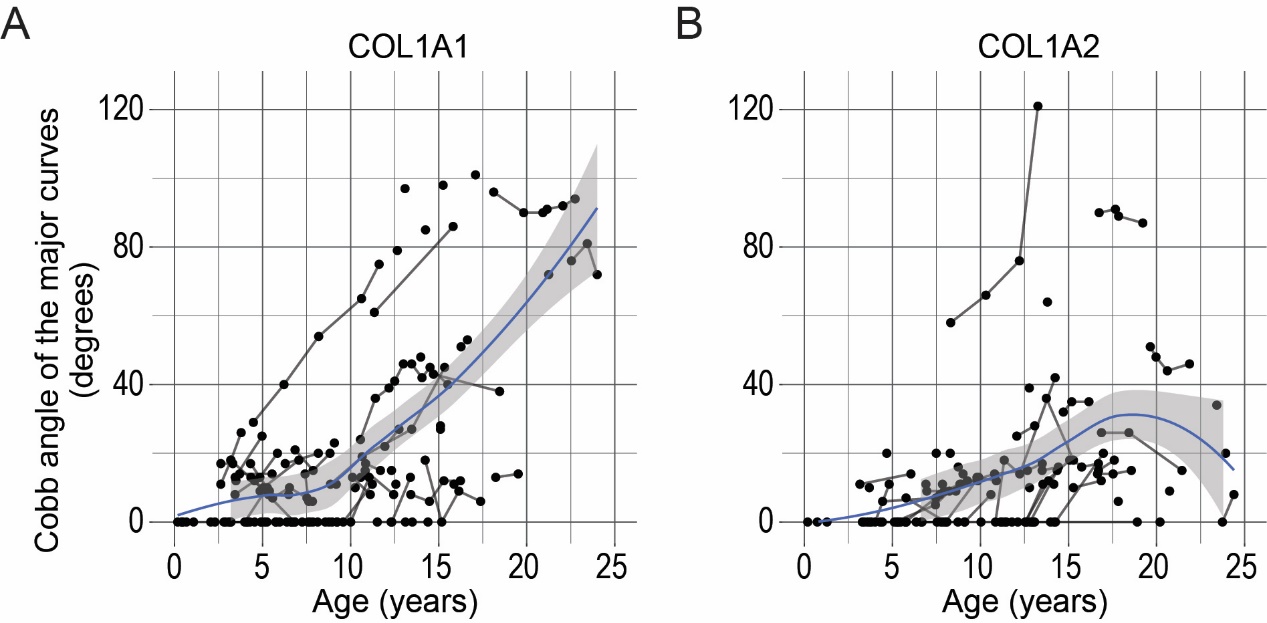
Figure S3** Progression rates of scoliosis in OI patients.

**A-B**, Cobb angles (degrees) of the major curve with respect to age, for patients carrying COL1A1 mutations (**A**) and COL1A2 mutations (**B**). Connected dots represent longitudinal follow-ups of the same patients. Connected dots represent longitudinal follow-ups of the same patients. Grey areas and medial curves are the standard error band and fitted values, respectively, using the LOESS model.
